# Superhydrophilic Interconnected Biomass‐Based Absorbers Toward High‐Speed Evaporation for Solar Steam Generation

**DOI:** 10.1002/gch2.202300046

**Published:** 2023-07-05

**Authors:** Dan Wei, Xiaoyu Cao, Miaomiao Ma, Zexiang Zhao, Jing Zhang, Xinyu Dong, Chengbing Wang

**Affiliations:** ^1^ School of Materials Science and Engineering Shaanxi Key Laboratory of Green Preparation and Functionalization for Inorganic Materials Shaanxi University of Science and Technology Xi'an Shaanxi 710021 China

**Keywords:** desalination, porous carbon materials, solar interface evaporation

## Abstract

Taking abundant and sustainable solar energy as the only energy source, solar‐powered interface evaporation has been regarded as a promising method to alleviate the pressure of freshwater shortage. However, the uptake of clean water from brine is constantly accompanied by evaporation of water and condensation of vapor, which inevitably generates salt solid, preventing further continuous and stable evaporation. The most direct method is to fabricate a photothermal material with salt self‐resistance by using the reflux of salt ions. Here, a superhydrophilic interconnected biomass carbon absorber (SBCA) is prepared by freeze‐drying and carbonization, realizing strong liquid pumping, and self‐blocking salt. In combination with superior broadband light absorption (94.91%), high porosity (95.9%), superhydrophilicity, and excellent thermal localization, an evaporation device with excellent evaporation rate (2.45 kg m^−2^ h^−1^ under 1 kW m^−2^) is successfully proposed. In the meantime, the porous skeleton and rapid water transport can enhance the diffusion of salt ions and slow down the rate of salt deposition. As a result, no salt deposition is found on the SBCA surface after continuous irradiation at 1 kW m^−2^ for 15 h. The design can provide a convenient and low‐cost efficient strategy for solar steam generators to address clean water acquisition.

## Graphical abstract

A superhydrophilic interconnected biomass‐based absorber (SBCA) for achieving high‐speed seawater desalination is fabricated. Due to the superior light‐thermal conversion and salt resistance, the SBCA can realize effective solar steam generation, which provides a new perspective of low cost, high efficiency, and environmental friendliness for solar desalination system.

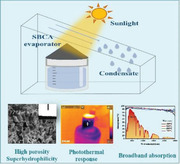

## 1 Introduction

The scarcity of clean water resources is considered as one of the bottlenecks that seriously restrict the sustainable and stable development of society.^[^ ^1^, ^2^, ^3^ ^]^ With the surge of population and accelerated urbanization, the problem of freshwater resources shortage will become more and more severe. Therefore, it is urgent to find a sustainable and efficient clean water production technology. Desalination is considered to be a promising solution. Currently, various water treatment technologies have been extensively studied,^[^ ^4^, ^5^ ^]^ such as multistage distillation (MED),^[^ ^6^ ^]^ electrodialysis,^[^ ^7^ ^]^ reverse osmosis membrane technology (RO),^[^ ^8^ ^]^ and ultrafiltration technology.^[^ ^9^ ^]^ However, as the concentration of brine increases, the pressure used to drive the filtration process in some mainstream technologies (e.g., RO) raises sharply, significantly shortening the lifetime of the membranes and leading to high water treatment costs along with environmental pollution and energy losses.^[^ ^10^, ^11^, ^12^, ^13^ ^]^ Therefore, desalination technologies that use green and nonpolluting solar energy as an energy source have emerged.

For the past few years, interface solar vapor generation (ISVG) has received much attention for its rapid evaporation and minimal carbon footprint in the treatment of saltwater or wastewater, overcoming the disadvantages of conventional solar desalination with high heat loss and low light absorption.^[^ ^14^, ^15^, ^16^ ^]^ In the process of solar seawater desalination, the optothermal conversion materials used for solar interfacial evaporation must meet the following three points: 1) outstanding sunlight absorption and thermal energy generation capacity;^[^ ^17^, ^18^, ^19^, ^20^ ^]^ 2) efficient thermal management to minimize heat loss;^[^ ^21^, ^22^, ^23^, ^24^ ^]^ 3) pore structure for steam to escape.^[^ ^25^, ^26^, ^27^, ^28^ ^]^ So far, photothermal materials such as biomass materials, carbon‐involved materials, metal plasma materials, semiconductor‐based materials, and porous polymers have been developed.^[^ ^29^, ^30^, ^31^ ^]^ Among them, a wide variety of plant structures have been used to develop solar steam generators due to their wide distribution, lower cost, and more friendly and simple preparation methods, which are more favorable for large‐scale production.^[^ ^32^, ^33^ ^]^ For instance, Wang et al. fabricated self‐floating super hydrophilic porous carbon foam (SPCF) by simply sintering potatoes in one step. It has ultrafast light‐thermal response and high porosity. An evaporation rate with 1.48 kg m^−2^ h^−1^ and photothermal conversion efficiency with 86% under 1 sun were obtained.^[^ ^34^ ^]^ Hu et al. innovatively proved that surface carbonized (SC) wood can be served as a solar vapor generator, and the light‐thermal conversion efficiency is as high as 80.4% (about 10 sun irradiation). It is attributable to the effective water transportation of wood and the high absorption of light on the carbonized surface.^[^ ^35^ ^]^ Then, various hierarchical structure designs were developed to meet the requirements of practical applications, which play an important role in improving the seawater evaporation rate and light‐thermal conversion efficiency. For example, Chen et al. prepared a carbon foam/exfoliated graphene double‐layer structure, and the photothermal conversion efficiency reached 87% under the sun illumination of 10 kW m^−2^. The top layer absorbs solar energy, while the bottom layer limits the thermal conduction to the bottom water.^[^ ^36^ ^]^ Furthermore, they developed a three‐layer ISVG device comprised of transparent bubble film/commercial ceramics/polystyrene foam to decrease convection, conduction and radiation heat losses, and achieved a solar absorber temperature of 100 °C and a solar thermal efficiency of 71% through unique heat regulation.^[^ ^37^ ^]^ In addition, the 1D and 2D wicking paths can manage the transportation of water well. By adjusting the water delivery process of the evaporator, a high‐performance ISVG device can be established. However, at present, the preparation process of multilayer structure is complex and the cost is high, so it is difficult to realize large‐scale production. Therefore, it is an urgent problem to design low‐cost ISVG devices with high evaporation rate through hierarchical structure.

During the generation of solar interfacial steam, the evaporator absorbs sunlight to generate heat, which heats the surface water layer, evaporates water and leaves salt ions at the interface. As time goes on, salt crystals will be deposited on the evaporator surface. However, white salt crystals will enhance the reflection of light, obstruct the overflow of steam, and deteriorate the performance of evaporator.^[^ ^14^, ^38^ ^]^ Therefore, it is essential for ISVG to develop an effective and salt‐tolerant vapor generator. At present, the salt mitigation strategies of interface solar evaporators are mainly divided into three categories, namely, zero liquid discharge strategy,^[^ ^39^, ^40^ ^]^ direct salt blocking strategy,^[^ ^41^, ^42^ ^]^ and salt ion reflux strategy.^[^ ^43^, ^44^, ^45^, ^46^ ^]^ Among them, salt ion diffusion reflux is regarded to be an easy and effective method to deal with salt accumulation. When salt crystals are deposited on the evaporator surface, the salt ion concentration on the evaporator surface raises dramatically. Due to different salt concentrations and chemical potential energy, crystallized solid salt will automatically diffuse into bulk water with relatively lower concentration. As we all know, the salt particles diffusion is closely related to the internal structure of evaporation device. The numerous pores channels in the evaporator are used for transporting water and diffusing salt ions. As a result, superhydrophilicity and rich pore channels are the key of salt ion reflux strategy. It should be noted that different shapes of 3D evaporator will exhibit different water transport capacities and salt gradients, and therefore will also obtain distinct evaporation performance. Much work on cylindrical evaporators has been reported,^[^ ^47^, ^48^ ^]^ for example, using sodium alginate as a substrate, Li et al. prepared a cylindrical solar steam generator with excellent salt resistance.^[^ ^49^ ^]^ Umbrella‐shaped, conical, and inverted conical evaporators have also gradually emerged in order to further increase the area of the evaporation surface. The results showed that the conical evaporator has faster water transport capability. Therefore, it would be meaningful if multiple shapes of evaporators could be prepared simultaneously from one material.

Here, a self‐blocking salt superhydrophilic interconnected porous biomass absorbers (SBCA) for efficient solar desalination was prepared by a simple carbonization process. The carbonized SBCA not only maintains the original porous structure of the raw material (95.9% porosity), but also achieves high light absorption capacity and superhydrophilicity. Specifically, the light absorption rate of SBCA can reach 94.91% in the full spectral range. At a simulated solar power of 1 kW m^−2^, the surface temperature of the dried SBCA could reach 70 °C within 30 s, showing an ultrafast photothermal response. Moreover, the difference between the upper surface temperature and the middle temperature of SBCA was as high as 20 °C after 30 min of light exposure, indicating the superb thermal management ability and thermal localization capability of SBCA. Integrated with ultralow thermal conductivity (0.06 W m^−1^ K^−1^ at 25 °C), the heat loss of SBCA during solar evaporation is greatly reduced. Moreover, as long as the water droplets contact the surface of SBCA, the water can be completely absorbed within 50 ms, and the contact angle is less than 5°. The highly open interconnection and large channel structure provide superb capillary forces, thus ensuring water transport and diffusive return of salt ions through the channels. As a result, the SBCA exhibits excellent capabilities in both rapid water circulation and superior salt resistance. Under one solar irradiation, the SBCA worked continuously for 15 h in 3.5 wt% simulated brine without visible salt particles on the surface. Furthermore, 0.5 and 1 g NaCl particles were added on the surface of SBCA. Under the illumination of 1 kW m^−2^, it took 3 h for 0.5 g NaCl particles to completely dissolve, and 4 h for 1 g NaCl to dissolve. This excellent salt discharge ability is of great significance to the long‐term stable operation of photothermal evaporator. In addition, by changing the shape of evaporator (cylinder, inverted cone, and cone), solar seawater desalination devices with different water transport capacity and evaporation performance were obtained, which expands the diversity of biomass evaporation devices and provides a new insight for the design of salt‐tolerant and efficient solar‐driven interface evaporation devices.

## 2 Results and Discussion

### 2.1 Fabrication and Characterization of SBCA

**Figure 1:**
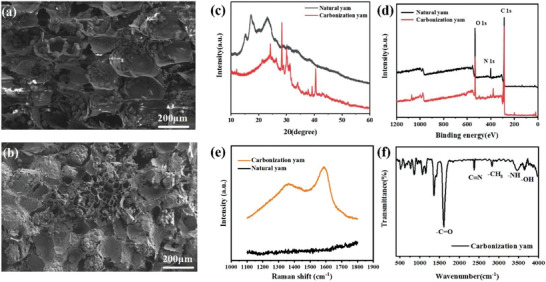
SEM images of a) freeze‐dried yam, and b) carbonization yam. c) XRD pattern, d) XPS spectrum, e) Raman spectrum, and f) FTIR spectrum of SBCA.

Superhydrophilic interconnected biomass carbon absorber (SBCA) was prepared by freeze‐drying and simple carbonization method using low‐cost and easily available yam as raw material, and the experimental procedure is shown in Figure S1 (Supporting Information). The yam was washed, cut into pieces, and freeze‐dried to remove water and retain the original pore structure. The morphology of the freeze‐dried yam is shown in **Figure** **1**a, with the pore size of about 130–160 µm. After carbonization at 400 °C, the volume of SBCA became slightly smaller and more interpenetrating pores were produced on the carbonized surface, which was mainly due to the negative pressure environment that allowed the sintering by‐products to escape completely, leaving more pore channels. In addition, the pore size of SBCA decreased to 70–110 µm after carbonization (Figure 1b). The presence of clustered particles on the surface can be clearly seen in the scanning electron microscope (SEM) images, and these rough structures can further increase the reflection and reabsorption of solar light and enhance the sunlight absorption. Moreover, a lot of pores can be found in the SEM images from the cross‐section of SBCA (Figure S2, Supporting Information), which provides diffusion pathways for rapid water transport and vapor overflow. From the X‐ray diffraction (XRD) pattern of SBCA (Figure 1c), we can see that the uncarbonated yam showed the standard cellulose d_101_ crystalline diffraction peak (12°), and exhibited the typical hemicellulose diffraction peak of pentosan within ≈18°–26°. After carbonization at 400 °C, the diffraction peak of hemicellulose is significantly weakened, which is mainly due to the cleavage of hemicellulose. Meanwhile, a sharp graphite diffraction peak is produced at 24°, which is attributed to the carbonization of cellulose into graphite. The graphite diffraction peaks are sharper and shifted toward higher angles, which implies the increasement of crystallinity and guarantees the high stability of SBCA. According to X‐ray Photoelectron Spectrometer (XPS, Figure 1d), SBCA contains carbon, nitrogen, and oxygen. Meanwhile, the peak splitting of N1s reveals the appearance of nitrogen‐containing groups on the surface of the carbonized sample (Figure S3a, Supporting Information), which plays an important role in improving the hydrophilicity of SBCA. The vibrations at 1608, 2371, 2803, 3472, and 3657 cm^−1^ in Fourier Transform Infrared Spectrometer (FTIR) correspond to —C=O, C≡N, —CH_3_, —NH, and —OH, respectively (Figure 1f). Among them, —C=O, —NH, C≡N, and —OH have good hydrophilicity, which further provides conditions for rapid water transport.

To further investigate the effect of carbonization temperature (300, 400, 500, 600, and 700 °C) on SBCA, the degree of graphitization of SBCA with different carbonization temperatures was investigated by Raman spectra. As shown in Figure 1e; and Figure S4 (Supporting Information), compared with natural yam. SBCA‐300, SBCA‐400, SBCA‐500, SBCA‐600, and SBCA‐700 all have 1350 cm^−1^ (D peak) and 1580 cm^−1^ (G peak), which indicates that the cellulose in SBCA are converted to graphite microcrystals. The *I*_D_/*I*_G_ ratios were 2.4, 2.39, 2.42, 2.49, and 3.6, respectively. At 400 °C, the degree of graphitization is higher. According to the ultradepth‐of‐field micrograph (Figure S3b–f, Supporting Information), the height differences between the highest and lowest points of the SBCA surface were 710.08, 461.08, 305.48, 362.63, and 196.48 µm, respectively. The surface height difference of SBCA‐300 was the largest, which might be caused by incomplete carbonization. And when the carbonization temperature reached 700 °C, the surface height difference of SBCA decreased significantly, which indicated the collapse of the internal structure. The results can reflect the surface roughness of SBCA from the side, and a rougher surface is more favorable for light absorption. Because the rough surface of SBCA is conducive to the formation of sunlight traps. It can promote the multiple reflection and refraction of sunlight, further enhancing the light absorption ability. Combined with the Raman test results, 400 °C is considered as the best carbonization temperature.

### 2.2 Water Transport Performance of SBCA

**Figure 2:**
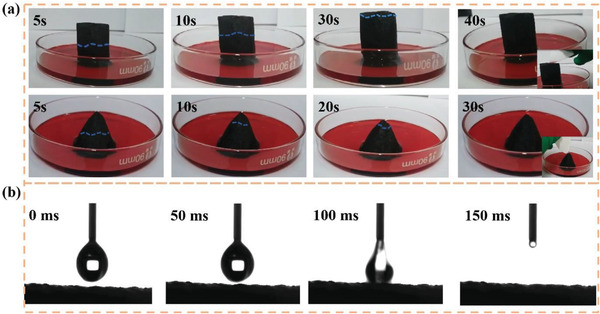
a) Water transport performance of cylindrical SBCA and conical SBCA. b) Contact angle measurement of SBCA.

Efficient water transport channel is a critical prerequisite for obtaining ultrahigh evaporation rate. Liquid pumping depends mainly on the capillary force of the pore. Since capillary pumping pressure is inversely proportional to the pore size, micrometer‐sized pores are commonly used for water pumping. The size of pores is particularly important for the water transport performance of SBCA. Too small a pore size tends to cause clogging during evaporation, while too large a pore size causes excess water to remain on the evaporating surface and consume heat. The water transport capacity of cylindrical SBCA and conical SBCA was examined by water transport experiments and contact angle tests. A 3 cm high dry sample was placed in red ink to observe the water transport position. As illustrated in **Figure** **2**a, the cylindrical SBCA and conical SBCA showed superior water transport ability by absorbing red ink to the upper surface in just 40 and 30 s, respectively. Figure S5 (Supporting Information) showed the contact angles of SBCA at different carbonization temperatures. Among them, due to incomplete carbonization of SBCA‐300, the tar produced from carbonization cannot be completely volatilized at too low temperature, but adhered to the surface, making the sample hydrophobic. With the increase of carbonization temperature, SBCA began to become hydrophilicity. When the temperature is 400 °C, as illustrated in Figure 2b; and Figure S5 (Supporting Information), the water droplets were completely absorbed by SBCA within 50 ms, and the contact angle was less than 5°, which was sufficient to prove the superhydrophilic characteristics of SBCA. However, although SBCA‐500 and SBCA‐600 were still hydrophilic, it took longer to absorb water droplets. In addition, after SBCA‐700 contacted with water for 1000 ms, there are still water droplets on the surface. Therefore, SBCA‐400 has superior water transport capacity. In fact, SBCA's strong pumping ability is mainly attributed to its unique pore structure and superhydrophilic characteristics. The open porosity and bulk density of SBCA were tested by Archimedes drainage method, and the results are illustrated in Table S1 (Supporting Information). The open porosity of SBCA‐400 reached 95.9%.

### 2.3 Photothermal Conversion and Interfacial Evaporation Performance of SBCA

**Figure 3:**
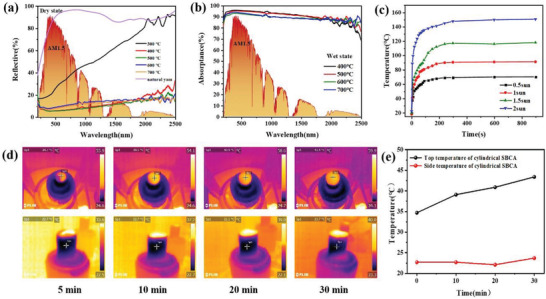
Photoresponse and thermal localization of SBCA. a) Reflective spectra of SBCA under dry conditions. b) Light absorption capacity of SBCA under wet conditions. c) Surface temperature of SBCA‐400 (dry) under different sunlight intensities. d) Infrared camera images of the upper surface and sides of the SBCA under 1 kW m^−2^ solar irradiation. e) The temperature comparison of the upper surface and the side surface of SBCA (cylinder).

In solar thermal evaporation, the sunlight absorption of absorber is of great significance to evaporation rate. Based on this, the reflectivity and absorbance of SBCA were tested by UV–visible‐near infrared spectrophotometer under two states of dry and wet, as shown in **Figure** **3**a,b. Under dry conditions, SBCA‐300 exhibited high reflection and low light absorption, which were not conducive to solar thermal evaporation. 92.06%, 92.32%, 88.65%, and 89.57% of light absorption were obtained for the dried SBCA from 400 to 700 °C, respectively, while under wet conditions, light absorptions were increased to different degrees (Figure S6a, Supporting Information). Among them, SBCA‐400 have the most superior light absorption rate of 94.91%. Then, the trend of the surface temperature of SBCA‐400 was observed under different simulated solar illumination, and the results are presented in Figure 3c. Under 1 kW m^−2^ solar illumination, the maximum surface temperature of SBCA‐400 rapidly increased to 80.5 °C within 90 s and finally stabilized at 81 °C. Under the light power of 2 kW m^−2^, the maximum surface temperature of SBCA increased dramatically from room temperature to 88.6 °C within 5 s. Due to the inhomogeneity of xenon lamp irradiation, the maximum and average temperatures in the surface temperature of SBCA‐400 were also measured under 1 sun irradiation. The temperature difference was kept within 4 °C as shown in Figure S6b (Supporting Information).

In addition, the solar absorber is often accompanied by different degrees of thermal loss during the water evaporation. Therefore, the ability of evaporator thermal management is very important to improve evaporation efficiency. Heat loss can be reduced through reasonable structural design and material selection. Therefore, polystyrene is used as a thermal insulation layer to lower heat conduction loss. At the same time, the thermal conductivity of porous yam carbon is as low as 0.06 W m^−1^ K^−1^ at 25 °C, which ensures the thermal insulation effect. To explain the thermal management ability of SBCA more intuitively, the temperature of the upper surface and side of cylindrical SBCA under 1 kW m^−2^ solar irradiation was recorded by FTIR. As illustrated in Figure 3d, with the extension of illumination time, the surface temperature of SBCA‐400 increased from 34.7 to 43.4 °C, but the temperature of the side surface did not increase significantly within 30 min, indicating that local hot spots appeared on the upper surface of SBCA‐400, and thermal localization was successfully realized. It can be seen from Figure 3e that after 30 min, the upper surface temperature of SBCA‐400 is much higher than that of the side surface, suggesting the superior heat management ability of SBCA.

**Figure 4:**
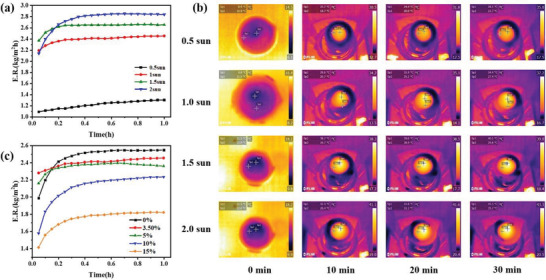
a) Evaporation rate of SBCA (cone) under different light intensities. b) Infrared camera images of SBCA (cone) under different light intensities. c) Evaporation rate of SBCA (cone) under different salt concentrations.

To cut down the material cost, conical SBCA evaporators were fabricated, which require only one‐third of the raw material of cylindrical SBCA, greatly reducing the cost of industrial mass production. Similar to the cylindrical evaporator, 2D radial water transport can greatly reduce heat loss, so the evaporative performance of the evaporator with inverted conical SBCA was measured by inverting the conical SBCA in simulated seawater (Figure S7, Supporting Information). And polystyrene foam was served as the support body and heat insulation layer. Under the irradiation of one sun, the evaporation rate of SBCA quickly reached a steady state, and the average value was calibrated by subtracting the inherent water evaporation in the dark field (Figure S8a, Supporting Information). As presented in Figure S8b (Supporting Information), the evaporation rate of inverted conical SBCA is only 1.8 kg m^−2^ h^−1^, which is lower than that of cylindrical SBCA (2.2 kg m^−2^ h^−1^, Figure S8c, Supporting Information). This is mainly because its side surface area is smaller than that of cylindrical evaporator, and the absorbed environmental energy is lower. At the same time, the cone tip directly contacts the water surface, and there is no direct thermal insulation layer, so the heat is more likely to diffuse into the water body. In order to address the problem of low evaporation rate of inverted conical SBCA, a conical evaporator placed forward was designed. Like the cylindrical evaporator, the evaporation rate of the conical SBCA device is 2.45 kg m^−2^ h^−1^ under 1 kW m^−2^ solar illumination (**Figure** **4**a), with polystyrene foam as the support body and thermal insulation layer and dust‐free cloth as the water transportation layer. The obvious enhancement of evaporation rate is mainly due to the fact that the conical evaporator has a larger surface area irradiated by light, and the relatively low surface temperature also reduces the environmental heat loss. The surface temperatures of the conical SBCA at different light intensities are shown in Figure 4b. When the light intensity is 2 kW m^−2^, the temperature in the hottest region of the conical SBCA surface increases from 11.5 to 42.4 °C within 10 min, and the temperature in the edge region also increases from 12 to 29.4 °C, which indicates that the conical sides can also receive light. At the same time, compared with cylindrical SBCA (Figure S9, Supporting Information) and inverted conical SBCA (Figure S10, Supporting Information), the slightly lower surface temperature of conical SBCA can reduce the loss of heat to the environment and achieve ultrahigh evaporation rate. Figure 4c shows the evaporation performance of conical SBCA in simulated seawater with different salinities. When the simulated sea salt concentration is 15 wt%, the evaporation rate can still reach 1.82 kg m^−2^ h^−1^, which is much higher than that of cylindrical evaporator (Figure S11a, Supporting Information) and inverted conical evaporator (Figure S11b, Supporting Information), providing a superior desalination performance for high salinity seawater. In addition, we also tested the evaporation rate of SBCA at different heights under 1sun irradiation (Figure S12, Supporting Information).

### 2.4 Salt Tolerance Performance and Cyclability of SBCA

**Figure 5:**
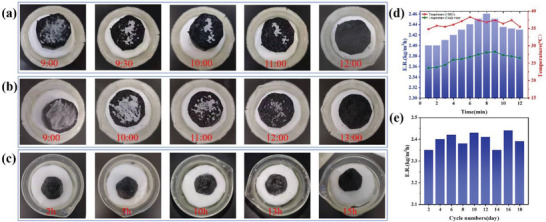
The self‐desalination and cyclability performance of SBCA. a) 0.5 g, and b) 1.0 g of NaCl were added on the surface of SBCA under 1 kW m^−2^ irradiation. c) Continuous solar interface evaporation using SBCA at 1 sun irradiation in simulated seawater. d) The recorded evaporation rate and surface temperature of SBCA operated continuously for 12 h. e) Cyclic test of SBCA.

Whether solar evaporation system can solve the surface salt deposition is a crucial problem to ensure the long‐term and efficient operation of evaporator. When salt particles are accumulated on the surface of evaporator, it is easy to block the overflow path of steam and increase light reflection, which deteriorates the performance of evaporator. The superhydrophilic property can realize rapid water circulation between internal water pathways. The salt crystals deposited on the surface layer diffuse into low‐concentration solution driven by the salt concentration difference, thus achieving the purpose of salt tolerance.^[^ ^15^ ^]^ For a more visual observation of the salt‐blocking ability, 0.5 and 1 g NaCl particles were placed on the surface of cylindrical SBCA with an surface area of 5 cm^2^, and the dissolution rate of salt particles was recorded under the irradiation of 1 sun. As shown in **Figure** **5**a,b, it took 3 h for 0.5 g NaCl particles to completely dissolve, and 4 h for 1 g NaCl to dissolve. The high concentration of brine on the evaporator surface is brought back to the water body below by rapid water transport to achieve effective salt reduction. Due to the powerful pumping ability and capillary force of the SBCA, there was no salt deposition on the evaporator surface after 15 h of continuous evaporation in the conical SBCA (under 1 sun and 3.5 wt% seawater) (Figure 5c). Then, SBCA worked continuously in seawater at 3.5 wt% salt concentration for 12 h. The results indicated that the evaporation rate increased slowly with time, reaching 2.46 kg m^−2^ h^−1^ after 7 h, and then a slow decrease occurred (Figure 5d). Here, the enhancement in evaporation rate is mainly attributable to the slow increase temperature of bulk water, while the decrease in evaporation rate is due to the gradual increase in salt concentration in the water column caused by continuous evaporation. In addition, the cycle stability of evaporator is very crucial for practical application. Therefore, under 1 sun irradiation, the evaporation rate of the conical SBCA evaporator was tested for 18 consecutive days when the salt concentration was 3.5 wt% (Figure 5e). The evaporation rate did not change obviously with increasing number of cycles. This phenomenon shows that SBCA evaporator provides excellent stability. In a word, the evaporator has stable desalination capacity and shows good potential in seawater desalination field.

### 2.5 Outdoor Solar Water Evaporation and Cyclability of SBCA

**Figure 6:**
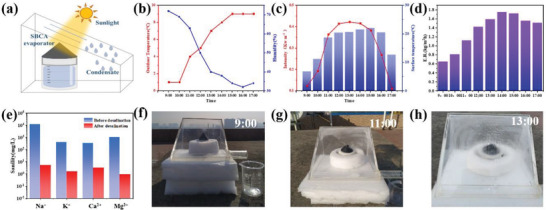
Outdoor water evaporation experiments using SBCA under natural sunlight. a) Schematic diagram of device in the outdoor experiments. b) Ambient temperature and humidity. c) Solar radiation intensities and surface temperatures of SBCA recorded from 09:00 to 17:00. d) Variation of evaporation rate of SBCA evaporator during 9 h of continuous outdoor desalination. e) Comparison of the concentrations of the four major ions in simulated seawater before and after solar desalination. Pictures of condensate collection device f) at 9:00, g) at 11:00, and h) at 13:00.

The condensate collecting device is illustrated in **Figure** **6**a. The data shown in Figure 6b,c are the results of ambient temperature, humidity, sample surface temperature and light intensity measured in outdoor experiments. In the test, the actual outdoor temperature was maintained at 1–9 °C, and the humidity began to decrease with the increase of temperature. Between 9: 00 and 17: 00, the maximum natural light intensity is about 0.431 kW m^−2^ at 13: 00, and the minimum light intensity is about 0.12 kW m^−2^ at 17: 00. Corresponding to the light intensity, the surface temperature of SBCA raised to 24.9 °C at 13:00. At lower outdoor temperature, the evaporation rate of SBCA reaches 1.75 kg m^−2^ h^−1^ (Figure 6d). In addition, the change of ion concentration of collected condensed water was traced by inductively coupled plasma spectrometry (ICP‐OES) to evaluate the solar seawater desalination ability of SBCA. The results show that the concentrations of Na^+^, K^+^, Ca^2+^, and Mg^2+^ in the condensation decrease rapidly. The ion concentrations are far below the World Health Organization (WHO) drinking water standard (Figure 6e). Figure 6f–h shows the actual water collection device, in which it can be clearly drawn that a large amount of condensed water is generated. The above outdoor measurements further indicate that SBCA has outstanding photothermal performance and possesses the potential to produce drinking water.

## 3 Conclusion

Solar‐powered interfacial desalination technology is becoming the key to break the water shortage dilemma. The development of evaporators with efficient solar thermal conversion properties and long‐term stable operation at low cost is a hot topic of current research. Here, we obtained superhydrophilic biomass carbon absorbers (SBCA) with excellent photothermal effect by carbonization process using biomass yam as raw material. SBCA has unique properties including broadband absorption (94.91%), high porosity (95.9%), superhydrophilic properties, low thermal conductivity (0.06 W m^−1^ K^−1^), and ultrafast thermal response (under the illumination of 1 sun, the surface temperature of dry SBCA reaches 70 °C within 30 s). Furthermore, superhydrophilic property of SBCA brings strong water pumping ability, and the porous interconnected microstructure makes the salt particles deposited on the surface redissolve, thus successfully achieving the purpose of salt reduction. After continuous operation for 15 h at 1 kW m^−2^ sun illumination, there was no salt aggregation on the evaporator surface. Moreover, the large evaporation surface and low heat loss allow SBCA to reach an evaporation rate of 2.45 kg m^−2^ h^−1^ under 1 kW m^−2^ light illumination. Since SBCA can guarantee effective photothermal conversion and self‐blocking salt with low cost, simple preparation process, and scalability, it has a guiding value in achieving the industrial production of salt resistance solar interface evaporation system.

## 4 Experimental Section

### Preparation of SBCA

The raw yam materials used for the experiments were obtained from a local supermarket. SBCA was prepared using a simple one‐step sintering method. The preparation process was divided into three main steps: preselection, pretreatment, and heat treatment. First, due to the irregularity growth of yam and possible damage caused by harvesting and transportation, to ensure the validity of the experiments, raw yams with uniform shape and no injury must be selected, washed, and peeled, cut into desired length and shape (cylinder, cone, and inverted cone), repeatedly washed and then frozen. Next, the frozen yam was placed in a freeze dryer for 48 h. Finally, SBCAs with different sintering temperatures (300, 400, 500, 600, and 700 °C) were obtained and washed repeatedly with deionized water and ethanol.

### Characterization of SBCA

SEM (FEI‐Apreo) was applied to observe the surface morphology of SBCA. The phase composition of SBCA was characterized by XRD (D8 advance) and the data were analyzed by MDI‐Jade 6 software. A Raman spectrometer (Renishaw inVia Reflex) with excitation wavelength of 532 nm was used to characterize the graphitization degree of the sample. X‐ray Photoelectron Spectrometer (XPS) (Thermo ESCALAB 250XI) was applied to determine the composition and chemical valence of porous SBCA. The groups on the sample surface were evaluated by FT‐IR spectroscopy (Vertex 70). The reflection spectrum of the light absorber in the wavelength range of 200–2500 nm was tested by the UV–visible‐near infrared spectrophotometer with the model Carry 5000. Further, the absorbance of SBCA can be calculated by the equation A = 1‐R‐T, where R and T represent the reflectance and transmittance of the sample, respectively.

### Self‐Desalination Experiment of SBCA

The SBCA was placed in simulated saltwater at a concentration of 3.5 wt% and evaporated continuously for 12 h. Subsequently, the deposition of salt particles on the absorber surface was observed. In addition, NaCl solids were directly sprinkled on the light absorption surface, and the salt removal performance of SBCA could be clearly observed after a period of time. Finally, the ions concentrations in the condensate were identified by ICP‐OES (Agilent 5110) to compare whether they comply the World Health Organization (WHO) potable water standards.

### Interfacial Solar Vapor Generation Experiment of SBCA

A xenon lamp equipped with AM = 1.5 filter was applied to simulate a solar light source, followed by salt water (3.5 wt%) to simulate seawater. A transparent glass beaker covered with thermal insulation polyethylene foam was employed as the water container. The evaporator was put on the surface of the simulated saltwater, the size of the simulated sunlight source was adjusted, and the sunlight intensity was recorded by an optical power density meter. An electronic precision balance and a computer were used to accurately record the mass change of water during evaporation (Figure S13, Supporting Information). Next, the evaporation rate ($\dot{m}$) was calculated using the following equation

(1) $$\dot{m}=m/st$$

where *m* represents the mass change during water evaporation, *s* represents the projected area of the sample, and *t* represents the evaporation time. The solar thermal conversion efficiency is evaluated by the following equation

(2) $$\eta =\dot{m}\;h_{\mathrm{lv}}/C_{\mathrm{opt}}q_{i}$$

where *h*_lv_ represents the phase transition enthalpy, *C*_opt_ is the optical concentration, and *q*_i_ represents the standard solar radiation (1000 W m^−2^).

## Conflict of Interest

The authors declare no conflict of interest.

## Supporting information

Supporting Information

## Data Availability

The data that support the findings of this study are available in the supplementary material of this article.
