# Supplementary material for: Superhydrophilic Interconnected Biomass‐Based Absorbers Toward High‐Speed Evaporation for Solar Steam Generation
Source: Glob Chall. 2023 Jul 5;7(9):2300046. doi: 10.1002/gch2.202300046 (PMC10517294; doi:10.1002/gch2.202300046)
Supplement: Supplementary file 1 — Supporting Information [file GCH2-7-2300046-s001.pdf]

Global Challenges
Open Access

Supporting Information

for *Global Challenges.*, DOI 10.1002/gch2.202300046

Superhydrophilic Interconnected Biomass-Based Absorbers Toward High-Speed Evaporation for Solar Steam Generation

Dan Wei, Xiaoyu Cao, Miaomiao Ma, Zexiang Zhao, Jing Zhang, Xinyu Dong and Chengbing Wang**

Supporting Information

Superhydrophilic Interconnected Biomass-based Absorbers Towards High-speed Evaporation for Solar Steam Generation

Dan Wei, Xiaoyu Cao, Miaomiao Ma, Zexiang Zhao, Jing Zhang, Xinyu Dong, Chengbing Wang**

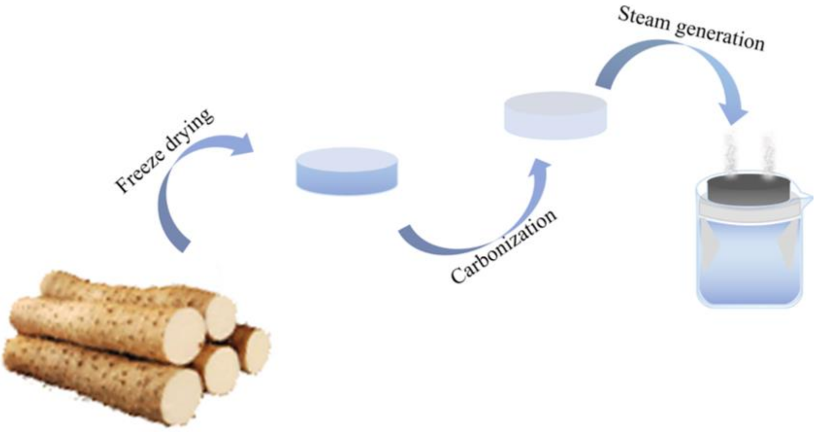

Figure S1. Schematic diagram of preparation of superhydrophilic biomass porous carbon-based evaporator.

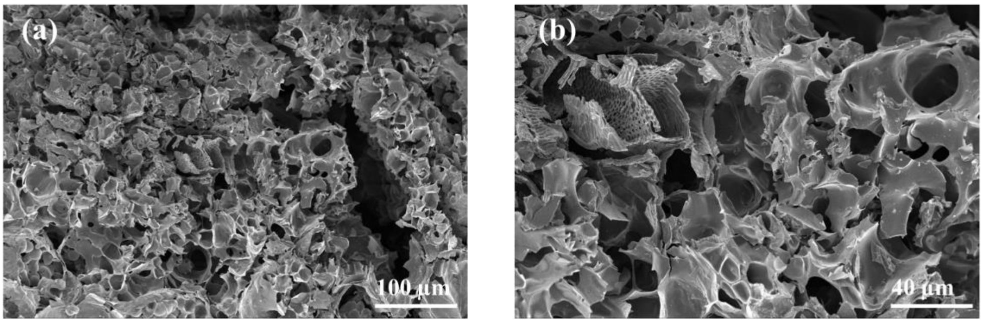

Figure S2. SEM images of the cross-section of SBCA with different resolutions.

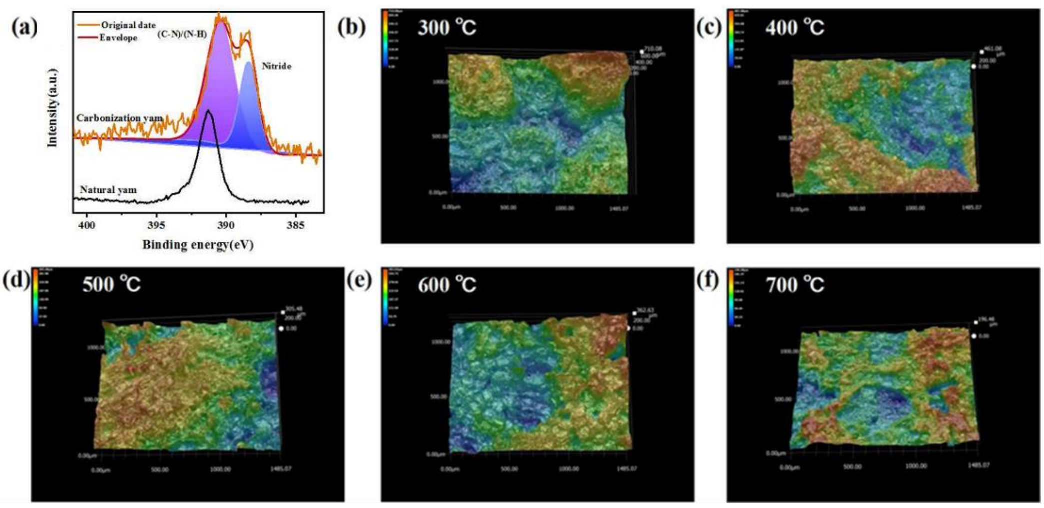

Figure S3. (a) Amplified map of N1s in XPS. Ultra-field micrographs of SBCA with carbonization temperatures of (b) 300°C, (c) 400°C, (d) 500°C, (e) 600°C, and (f) 700°C.

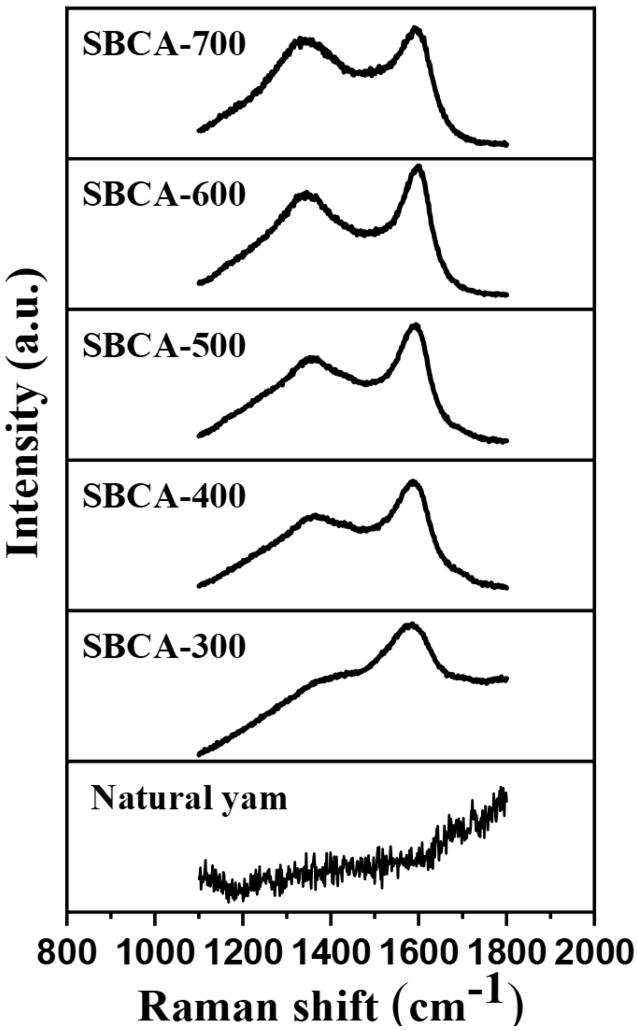

Figure S4. Raman spectra of natural yam and yam with different carbonization temperatures.

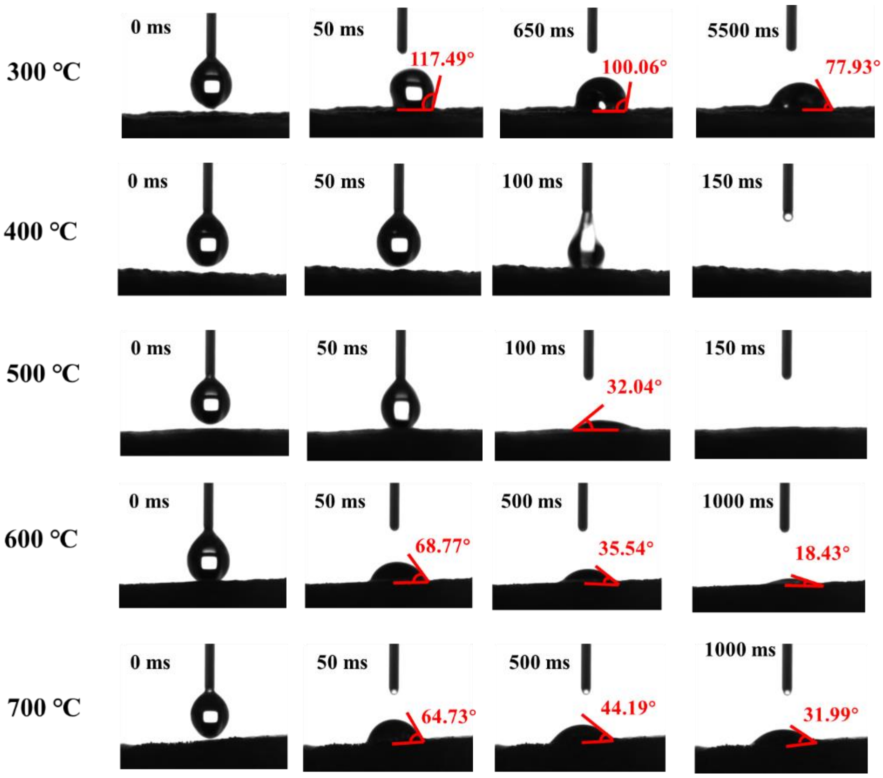

Figure S5. Contact angle measurement of SBCA under different carbonization temperatures.

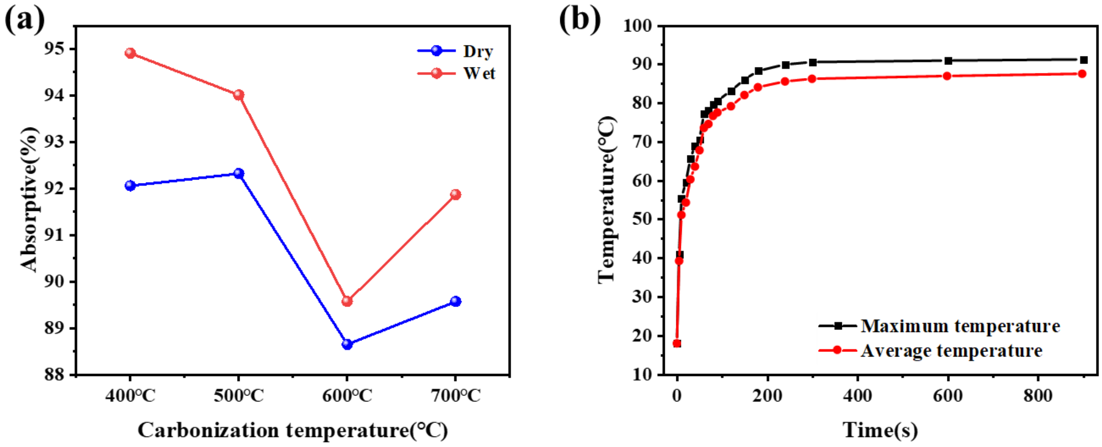

Figure S6. (a) Light absorption rate comparison chart under different carbonization temperature. (b) Comparison of the maximum and average temperature of the SBCA surface under sun irradiation.

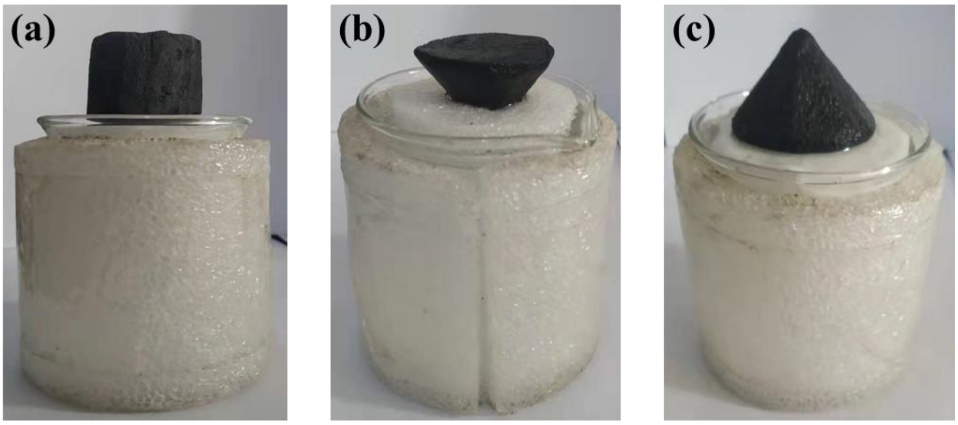

Figure S7. Photos of (a) cylindrical SBCA, (b) inverted conical SBCA, and (c) conical SBCA.

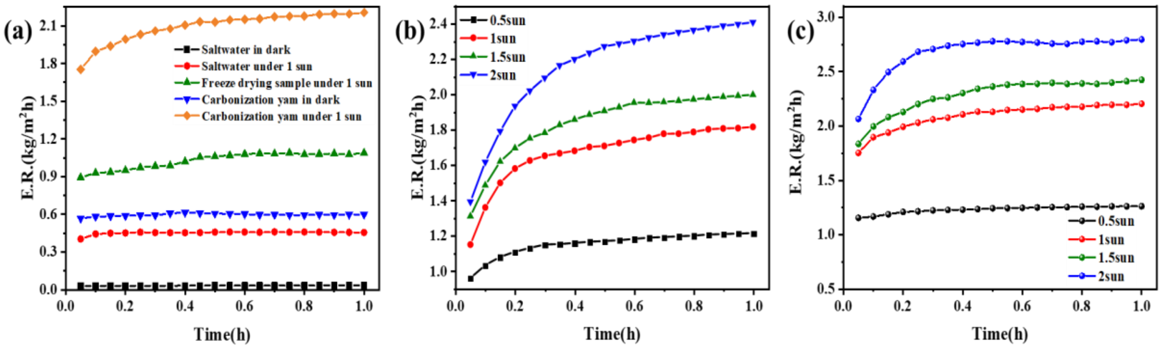

Figure S8. Evaporation rate of (a) carbonization yam, saltwater, and freeze-drying sample under different conditions, (b) cylindrical SBCA, and (c) inverted conical SBCA under different light intensities.

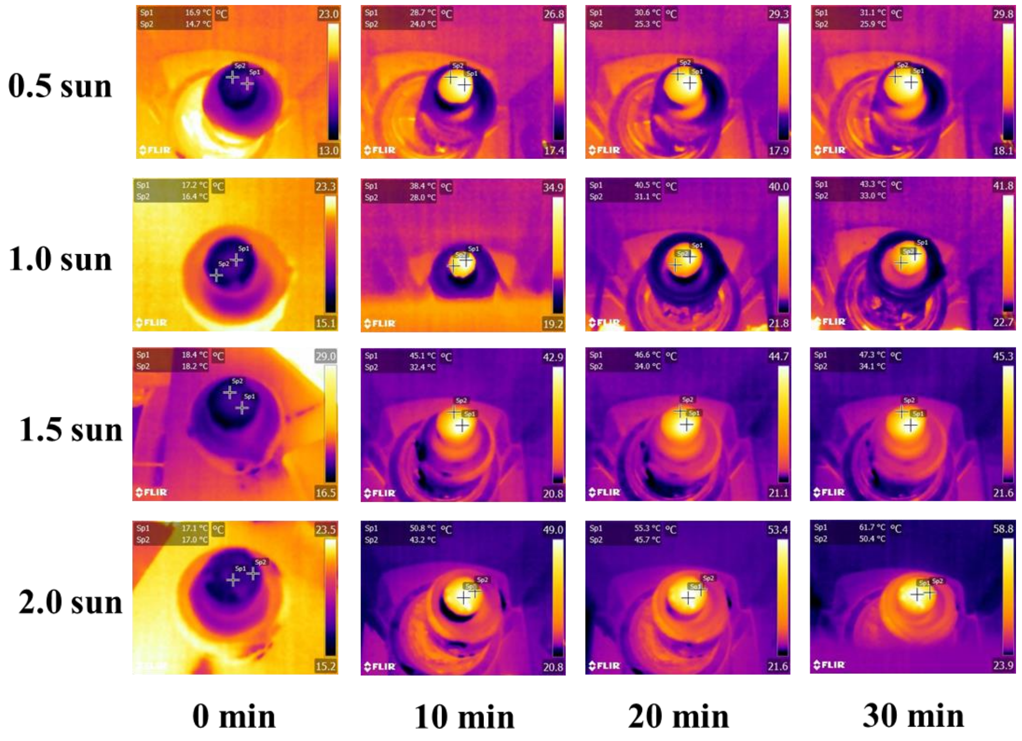

Figure S9. Evaporation rate of cylindrical SBCA under different light intensities.

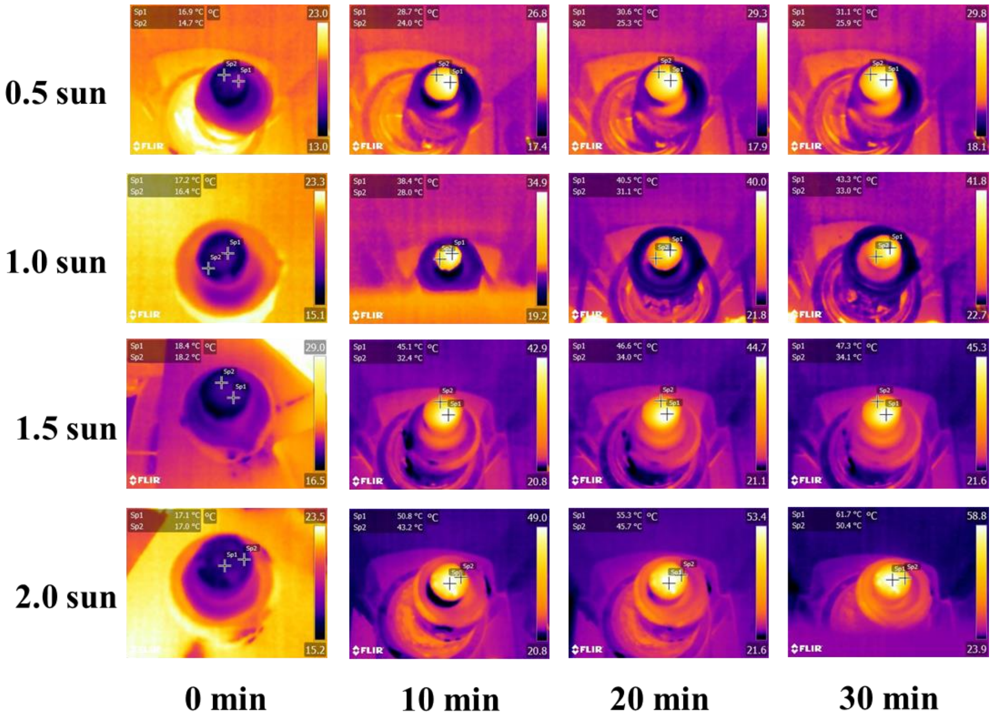

Figure S10. Evaporation rate of inverted conical SBCA under different light intensities.

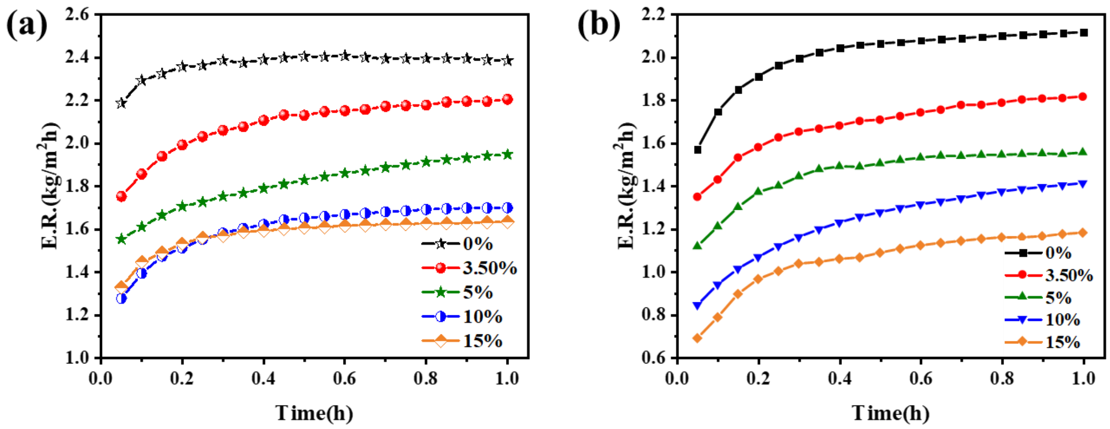

Figure S11. Evaporation rates of (a) cylindrical SBCA, and (b) inverted conical SBCA under different salt concentrations.

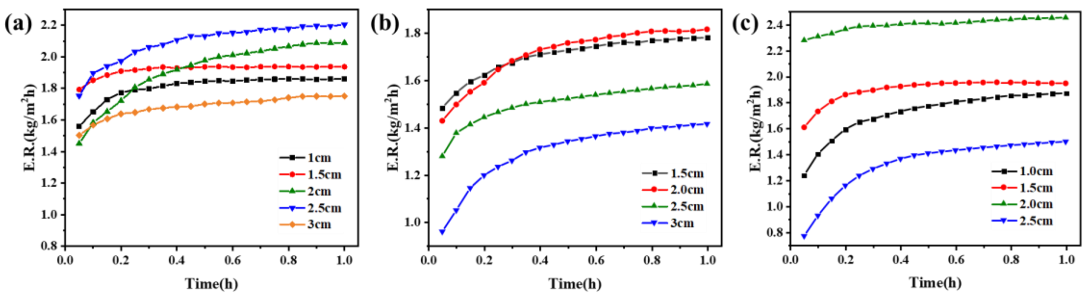

Figure S12. Evaporation rate of (a) cylindrical SBCA, (b) inverted conical SBCA, and (c) conical SBCA at different heights under 1 sun irradiation.

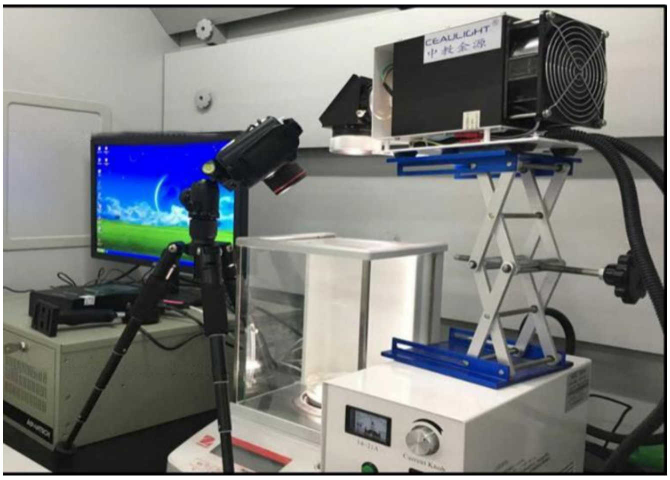

Figure S13. Diagram of the solar evaporation experiment device.

Table S1. Open porosity and bulk density of SBCAs at different carbonization temperatures.

Sample	Open porosity	Bulk density
SBCA-300	92.6%	10.3%
SBCA-400	95.9%	12.5%
SBCA-500	89.3%	10.2%
SBCA-600	96.2%	15.8%
SBCA-700	90.5%	11.1%
